# CT radiation profile width measurement using CR imaging plate raw data

**DOI:** 10.1120/jacmp.v16i6.5512

**Published:** 2015-11-08

**Authors:** Thorarin A Bjarnason, Chang‐Ying Joseph Yang

**Affiliations:** ^1^ Diagnostic Imaging Services Interior Health Kelowna BC Canada; ^2^ Department of Radiology University of British Columbia Vancouver BC Canada; ^3^ Department of Computer Science Mathematics, Physics & Statistics, University of British Columbia Okanagan Kelowna BC Canada; ^4^ Sindi Ahluwalia Hawkins Centre for the Southern Interior British Columbia Cancer Agency Kelowna BC Canada

**Keywords:** computed tomography (CT), collimation, computed radiography (CR), quality control, radiation profile, FWHM, MATLAB

## Abstract

This technical note demonstrates computed tomography (CT) radiation profile measurement using computed radiography (CR) imaging plate raw data showing it is possible to perform the CT collimation width measurement using a single scan without saturating the imaging plate. Previously described methods require careful adjustments to the CR reader settings in order to avoid signal clipping in the CR processed image. CT radiation profile measurements were taken as part of routine quality control on 14 CT scanners from four vendors. CR cassettes were placed on the CT scanner bed, raised to isocenter, and leveled. Axial scans were taken at all available collimations, advancing the cassette for each scan. The CR plates were processed and raw CR data were analyzed using MATLAB scripts to measure collimation widths. The raw data approach was compared with previously established methodology. The quality control analysis scripts are released as open source using creative commons licensing. A log‐linear relationship was found between raw pixel value and air kerma, and raw data collimation width measurements were in agreement with CR‐processed, bit‐reduced data, using previously described methodology. The raw data approach, with intrinsically wider dynamic range, allows improved measurement flexibility and precision. As a result, we demonstrate a methodology for CT collimation width measurements using a single CT scan and without the need for CR scanning parameter adjustments which is more convenient for routine quality control work.

PACS numbers: 87.57.Q‐, 87.59.bd, 87.57.uq

## I. INTRODUCTION

Measuring the computed tomography (CT) collimation width is a straightforward quality control measure that impacts both image quality and patient dose. Many jurisdictions and accreditation agencies require the profile to be assessed at acceptance^1^, ^2^, ^3^ and often routinely.^2^, ^3^ Most CT facilities have access to computed radiography (CR) systems and performing CT collimation profile assessments using CR requires no additional equipment or consumables, unlike film alternatives.

Previous studies have shown how to effectively use CR plates to measure the radiation profile width at the isocenter of the CT gantry.^4^, ^5^ These studies compared CR plate measurements with Gafchromatic film, ionization chambers, and X‐ray film and found that CR can be used to accurately measure CT collimation width. More recently, CR plates were used to assess CT table feed speed.^(6)^

A limitation of the previous work is that some CR systems reduce the digital dynamic range of the images to 10 bits for processed image data stored in Digital‐Imaging‐and‐Communications‐in‐Medicine (DICOM) image format, requiring the CR processor settings to be adjusted to prevent clipping of the CT radiation profile data according to the CT technique used.^(4)^ Clipping of the radiation profile hinders the ability to measure CT collimation width because the full width at half maximum (FWHM) of the penumbras cannot be found if the maximum intensity is unavailable. In addition, when the bit‐depth is reduced to avoid clipping, the precision of the half maximum locations can be affected. Even if proper adjustments are made to CR processing, nonlinear tone scaling in the processed data might still be present.

In this work we demonstrate how raw FujiFilm CR image data can be used for routine CT collimation width measurements. The quality control scripts are shared as an open source project named ‘X‐ray QC’ on sourceforge.org.

## II. MATERIALS AND METHODS

### A. Establishing the relationship between CR raw data pixel value and air kerma

The grey‐scale‐to‐air‐kerma mapping of CR image raw data was assessed using projection radiography with a similar approach as Liu et al.^(4)^ A 3 mm thick lead plate with a 3 cm square hole was placed on the CR plate and, using a radiography system, the light field collimated down to the size of the hole. The lead plate was moved around the surface of the same CR plate for each exposure of different levels, ensuring at least 9 cm spacing between the exposed areas, resulting with a grid of different, known, air kerma values on the same CR plate. The cells in the grid were exposed to a range of air‐kerma values between 37−1236 μGy at 80 kVp as determined by a dosimeter (model Xi with R/F detector, Fluke Biomedical/Raysafe, Billdal, Sweden) that is calibrated annually. Once processed by the CR scanner, the resulting raw image data consisted of a grid of regions with pixel intensities corresponding to known air‐kerma values. Average grey‐scale values within each exposed square were measured from the raw image plate using ImageJ (National Institute of Mental Health, Bethesda, MD) and plotted against the measured air kerma values.

For FujiFilm (Tokyo, Japan) image raw data, the following equation, based on previous work,^4^, ^7^ was used to relate air kerma to pixel value:

(1) $$y=1024\;{\text{log}}_{10}(x)+C$$

where *y* is the raw pixel value, *x* is the detector air kerma in μGy, and *C* is a scalar. The pixel value that corresponds with half of the maximum intensity of the CT radiation profile is as follows:

(2) $$y_{h}=y_{\text{max}}-1024{\text{log}}_{10}(2)$$

The equation to find the pixel value for $y_{h}$ does not depend on *C* and the pixel value of $y_{max}$, which corresponds to the maximum intensity of the CT radiation profile, is required.

### B. CT collimation assessment using CR raw data

Two models of Fuji CR systems were used for this analysis depending on availability: FCR XF‐1 and FCR Capsula XLII. For all experiments, FujiFilm type C cassettes with Fujifilm ST‐VI 35×43 cm cm photostimulable phosphor plates were used, which have a resolution of 5 pixels/mm (pixel width=0.2 mm).^(8)^

Collimation width was assessed for the CT scanners listed in Table 1. Fourteen CT scanners were used, one from nuclear medicine, two from radiation therapy, and eleven from diagnostic imaging departments.

**Figure 1:**
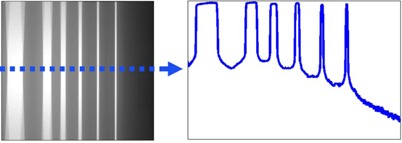
An example of a CR plate scanned using multiple CT collimations, advancing the table between each scan (left, gray scale). The pixel values down the center of the CR plate (dashed blue line) gives a profile from which collimation widths are measured (right).

The CR plate was placed on the patient bed with the lead side down and leveled, and the table was adjusted and aligned with the positioning lasers so the CR cassette plane was at isocenter in the CT gantry. This way of positioning of the CR plate at CT gantry isocenter is crucial to ensure the image of the collimation profile under an axial scan is parallel to the image pixel grid and the narrowest profile width is located near the center of the CR plate. Several collimations were measured on a single CR plate by moving the table a sufficient amount between acquisitions without overlapping by using spacings at least twice the prescribed collimation width. All available collimations for each scanner were assessed. An example of the resulting raw CR image from CR plate exposure to all available CT collimations from one scanner is shown in the left side of Fig. 1. CR plates were left inside the cassettes for these measurements since the study by Liu et al.^(4)^ showed that the minimal scatter from the CR cassette caused no changes in the FWHM. The following CT scan parameters were used for most collimation measurements: 80 kVp, 10 mA, 1 s tube rotation. The SPECT/CT scanner used 80 kVp, 30 mA, 0.5 s tube rotation, because these were the parameters available on the machine closest to those used on all other CTs.

MATLAB (R2013a, MathWorks, Natick, MA) was used to develop scripts to analyze raw CR image data having the *.std extension for Fuji CR systems (FujiFilm). Microsoft Windows Explorer is available on the CR scanner computer and can be used to search for the raw image data file folder, and contents were sorted by "Date modified". Using the open source MATLAB scripts, raw CR image data were opened and a profile was created, as shown on the right side of Fig. 1, by using the pixel values extracted from the central row of the CR image matrix, as demonstrated with the dashed blue line on the left side of Fig. 1. This central row of pixels is perpendicular to the CT radiation profile and captures the narrowest region of the CT radiation profile, because the CR cassette was positioned at the isocenter of the CT gantry. From this profile, the FWHM of each peak was measured as the collimation width at the CT scanner isocenter. Since the resolution of the CR plates used was 0.2 mm, the error in the measurements was 0.4 mm. The scripts are released as part of the open source project ‘X‐ray QC’ on sourceforge.org^(9)^ with Creative Commons licensing.^(10)^

**Table 1:** Breakdown of scanners participating in this study

*Vendor*	*Model*	*Total*
General Electric	LightSpeed VCT	3
General Electric	Optima CT660	1
General Electric	Discovery 750 HD	2
General Electric	Lightspeed Ultra	2
General Electric	LightSpeed R16	2
Philips	Precedence SPECT/CT	1
Siemens	Definition Flash	1
Toshiba	Aquilion 64	2

The resulting measured collimations were used to assess the scanners in accordance to the limits recommended in the ACR CT Quality Control Manual: "the measured radiation beam width should be accurate to within 3 mm or 30% of the total nominal collimated beam width, whichever is greater".^(3)^

### C. Comparison with established methodology

A similar approach to Liu and colleagues^(4)^ was used for both CR reader models using all available CT collimations from three of the scanners listed in Table 1. The following equation, similar to Eq. (2) and based on the work by Liu et al., was used to find the half maximum values used to measure the FWHM:

(3) $$y_{hD}=y_{\text{max}D}-1024/L\;{\text{log}}_{10}(2)$$

where *D* represents gray‐scale values from CR processed image data, and *L* is the latitude of the CR processor algorithm that controls the slope of the amplifier response.^(7)^ The raw data from the same IP plates were analyzed using the approach outlined above and the results were compared.

## III. RESULTS

### A. Establishing the relationship between CR raw data pixel value and air kerma

**Figure 2:**
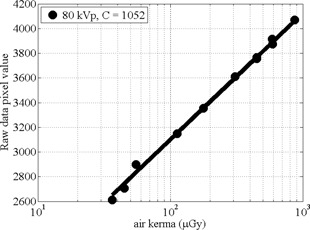
CR plate mean raw pixel values from different air kerma using 80 kVp radiographic X‐rays. The pixel value mapping plateaus at 12 bits (4095 ‐ not shown) for air kerma >935μGy.

A log‐linear relationship with air kerma and raw CR pixel value was confirmed, with C=1052, as shown in Fig. 2. However, the pixel value corresponding to half of maximum intensity only requires a log‐linear model without knowing C, as long as the CR plate image data are not saturated. Therefore, the MATLAB scripts will work with any kVp if the log‐linear relationship can be confirmed, since only C would change with kVp.

It was found that air kerma >935μGy saturated the CR plate resulting with raw data pixel values of 4095, which corresponds to the maximum value of 12 bit ($2^{12}$) data range.

### B. CT collimation assessment using CR

**Figure 3:**
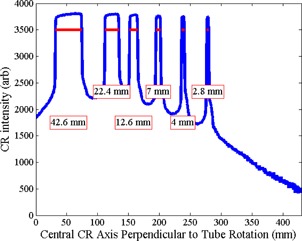
CT collimation width measurements from a GE 660 scanner using the MATLAB scripts developed for Fuji CR raw image data. The red horizontal lines show where the width measurements were taken and the boxes contain the measured collimations.

**Figure 4:**
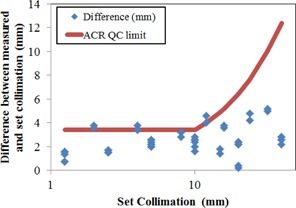
Difference between set collimations widths and measured collimation widths (diamonds), along with ACR QC limit of 3 mm or 30%, whichever is greater (line), using raw CR image data.

Example collimation measurements for a GE 660 CT are shown in Fig. 3. The red horizontal lines represent the FWHMs. The measured collimation widths from all scanners listed in Table 1 are compared with the ACR CT Quality Control Manual limits^(3)^ in Fig. 4. In total, 11 collimation measurements from four different scanners exceeded the ACR limit. For this study, only scanners from one vendor were consistently within ACR specifications.

### C. Comparison with established methodology

A total of 18 collimations were assessed from three CT scanners ranging from 1.25 to 40 mm. The CR processed DICOM CT collimation widths measured either 0, 0.2, or 0.4 mm larger than those found using the raw data approach from the same scans. These differences were found to be 11%, 33%, and 56% of the 18 measurements, respectively, with the specific breakdown shown in Table 2.

**Table 2:** A breakdown of the difference in collimation width measurements using processed and raw CR data. The processed CR data was either 0, 1, or 2 pixels wider, corresponding to 0, 0.2, or 0.4 mm, respectively, compared to the raw CR data

	*Difference*		
*Collimation*	*0 mm*	*0.2 mm*	*0.4 mm*
40 mm	0	1	*2*
20 mm	0	0	3
10 mm	0	2	1
5 mm	1	2	0
2.5 mm	1	0	2
1.25 mm	0	1	2

## IV. DISCUSSION

This technical note demonstrates the application of CT collimation width measurements as part of routine quality control for diagnostic imaging, radiation therapy, and nuclear medicine departments, spanning four vendors. The health region used in this study spans 220,000 sq km and all the CR readers have networked drives within the region's Intranet. Once the CR imaging plates were read, the raw data were sent to a network folder for analysis. There were nine CR readers, in geographically dispersed hospitals throughout the health region, used in this study, serviced by five different individuals. Using raw data from CR readers circumvented the need to confirm, adjust if necessary, and maintain the adjusted sensitivity profile settings required for analyzing CR processed DICOM data.

When comparing the raw data analysis approach with an established method using CR processed images with reduced bit depth, the processed collimation width measurements were either 0, 0.2, or 0.4 mm wider than those found using the raw data approach. These deviations were expected when considering the dynamic range of the DICOM data was reduced from 12 bits to 10 bits by the CR reader. Depending on how numbers were rounded during bit depth reduction, the precision of the half maximum measurement of the penumbras can be affected. A difference of 0 mm means neither penumbra was affected, while 0.2 mm means that rounding affected the half maximum determination of one of the penumbras, and 0.4 mm means both penumbras were affected. These discrete measurements represent 0, 1, and 2 pixel differences. Consequently, using raw CR data at worst is as accurate as CR processed reduced bit data and, at best, is 0.4 mm more precise for the imaging plates used here.

A limitation of the reduced bit depth approach is accidental signal clipping. For instance, when performing the collimation acquisitions on certain CT scanners, in specific cases when the CT protocol is adjusted to reduce the collimation by half the console automatically doubles the tube current. If not caught at the CT console during scan time, even if the CR reader adjustments are made according to Liu et al.,^(4)^ it is possible to have some clipped collimation data. Analyzing the raw data reduces the risk of acquiring the wrong data and the need to repeat the test with additional time and traveling cost. It is the raw data format that should have been used if it is available. The method of careful selection of the proper S and L values to avoid saturation from the processed image was developed only because the availability of the raw data was not made known to many physicists.

A limitation of this work is that only one CR vendor was used. However, we anticipate that this approach will work equally well using other CR vendors if raw data are available. Most, if not all, CR systems follow a log‐linear model mapping radiation intensity to raw data pixel value, and the approach outlined for confirming this behavior and the MATLAB scripts provided in this technical note can be used for CT collimation width measurements. The source code is released using a Creative Commons attribution, share alike version 3.0 unported licensing^(10)^ on soureforge.org under the X‐ray QC project.^(9)^ This licensing allows users to freely copy and distribute the scripts, and to further develop the code, as long as there is attribution and modified versions are distributed using the same license.

Two vendors had a single collimation width that exceeded the ACR limits, but they were for collimations rarely used on the scanners clinically, so no follow‐up was performed. One vendor consistently failed for smaller collimations, and upon follow‐up it was discovered that this vendor follows a factory default limit of 5 mm from the nominal value for all collimations. The vendor has been given the results from this study and is aware of the discrepancy between its collimation limits and the ACR guidelines.

In this work we built on previous studies that show CR can be used for assessment of radiation profile for CT scanners.^4^, ^5^ By establishing the log‐linear relationship between radiation intensity and raw pixel value, the FWHM can be determined using one scan, which is an improvement compared to the approach used by Jackson et al.,^(5)^ where two scans were required, one with half the dose of the other. Raw CR data, which has an intrinsically larger dynamic range than CR processed data, allow for improved measurement precision. Careful CR reader sensitivity adjustments, such as those suggested by Liu et al.,^(4)^ are not required for routine CT collimation measurements when using raw CR data.

## ACKNOWLEDGMENTS

Special thanks to the Biomedical and IT staff for CR reader maintenance and networking.

## Supporting information

Supplementary Material

Supplementary Material
